# Evaluating Helicobacter pylori as a Risk Modifier for Barrett’s Esophagus in Gastroesophageal Reflux Disease: Insights From a Multicenter Electronic Health Record-Based Study

**DOI:** 10.7759/cureus.95962

**Published:** 2025-11-02

**Authors:** Elizabeth Beyene, Mekdem Bisrat, Yonas Fetle, Syed F Gillani, Sair Ahmad Tabraiz, Addishiwot T Wudeneh, Daniel A Larbi, Miriam Michael

**Affiliations:** 1 Internal Medicine, Howard University Hospital, Washington, D.C., USA; 2 Internal Medicine, Marshall University Joan C. Edwards School of Medicine, Huntington, USA; 3 Pathology, St. Paul Millennium Medical College, Addis Ababa, ETH

**Keywords:** barrett’s esophagus, gastroesophageal reflux disease (gerd), gerd, helicobacter pylori, real-world data, trinetx global collaborative network

## Abstract

Background: The association between *Helicobacter pylori* (*H. pylori*) infection and the development of Barrett’s esophagus (BE) in patients with gastroesophageal reflux disease (GERD) remains controversial. This study aimed to assess the impact of BE among GERD patients with and without evidence of *H. pylori* using a large real-world dataset.

Methods: A retrospective cohort analysis was conducted using the TriNetX Global Collaborative Network comprising 147 healthcare organizations. Two cohorts of patients aged 18-50 years with GERD were identified: Cohort 1 (n=179,757), GERD patients with confirmed *H. pylori *infection, and Cohort 2 (n=179,757), GERD patients without *H. pylori *exposure. Propensity score matching was applied to balance demographics and comorbidities. The primary outcome was the diagnosis of BE (based on International Classification of Diseases, Tenth Revision (ICD-10): K22.7). Risk, survival, and incidence analyses were performed using measures of association, Kaplan-Meier curves, and t-tests.

Results: The incidence of BE was significantly higher in the GERD with *H. pylori *group (0.66%) compared to the GERD-only group (0.09%) (risk ratio (RR): 7.30, 95% CI: 6.20-8.60; *p*<0.001). Kaplan-Meier survival analysis demonstrated lower survival probability free of BE in the *H. pylori* cohort (97.84% vs 99.40%; log-rank p<0.001), with a hazard ratio (HR) of 7.13 (95% CI: 6.05-8.40; p=0.029). The number of BE instances was also significantly greater in the *H. pylori *cohort (mean: 3.15 vs. 1.67, p<0.001).

Conclusions: Among young adults with GERD, concomitant* H. pylori *infection is associated with a significantly higher risk of developing BE. These findings challenge the previously held notion of a protective role of *H. pylori* and suggest the need for targeted surveillance in this subgroup.

## Introduction

Barrett’s esophagus (BE) is a premalignant condition characterized by the replacement of normal squamous epithelium in the distal esophagus with specialized intestinal metaplasia due to chronic gastroesophageal reflux disease (GERD). It significantly increases the risk of developing esophageal adenocarcinoma, with annual progression rates reported between 0.12% and 0.5% [1,2]. While BE remains asymptomatic in many individuals, its clinical significance lies in the need for surveillance and early detection to mitigate cancer risk. Risk factors include chronic GERD, male sex, age over 50 years, central obesity, and smoking, with GERD being the most strongly associated [2].

*Helicobacter pylori* (*H. pylori*) is a gram-negative bacterium that colonizes the gastric mucosa and is known to cause chronic gastritis, peptic ulcer disease, and gastric malignancies [3]. The World Health Organization (WHO) has classified *H. pylori* as a Group 1 carcinogen, leading to gastric adenocarcinoma [4]. Another neoplastic disease caused by chronic *H. pylori* infection is gastric mucosa-associated lymphoid tissue lymphoma (MALToma), a condition that is much less common than peptic ulcer disease or gastric adenocarcinoma [5]. *H. pylori *infection is typically diagnosed via noninvasive tests such as the urea breath test, stool antigen test, and serology, or through invasive methods like endoscopic biopsy with rapid urease testing, histology, or culture [6].

*H. pylori* has been extensively studied in relation to upper gastrointestinal pathologies, yet its role in esophageal conditions remains controversial. Some studies have proposed a protective effect of *H. pylori*, particularly cagA-positive strains, which may reduce the risk of developing BE by suppressing gastric acid secretion and altering the gastric environment [7,8]. However, findings across studies remain inconsistent, and the relationship between *H. pylori* and esophageal pathology is complex and not fully understood. The interplay between protective and harmful mechanisms complicates our understanding of *H. pylori’s* role in BE. This study aims to evaluate whether the presence of *H. pylori* infection in patients with GERD is associated with an increased incidence of BE, using a large, matched cohort to clarify the clinical relevance of this association.

## Materials and methods

Study design

This retrospective cohort study was conducted using the TriNetX Global Collaborative Network, which aggregates de-identified electronic medical records from 147 healthcare organizations worldwide [9]. The study focused on adult patients aged 18 to 50 years with a diagnosis of GERD and aimed to assess the association between *H. pylori* infection and the subsequent development of BE.

Two cohorts were defined based on the presence or absence of *H. pylori*: Cohort 1 (GERD and *H. pylori*) which included patients aged 18-50 with GERD and confirmed *H. pylori* infection identified through the International Classification of Diseases, Tenth Revision (ICD-10) codes, laboratory results (e.g., urea breath test, stool antigen, or serum antibody), or relevant procedural codes [10]. Patients with any prior diagnosis of BE, esophageal malignancy, varices, ulcer, obstruction, achalasia, or history of esophageal surgery (fundoplication or esophagectomy) were excluded. Patients older than 50 years were excluded, as advancing age is a known risk factor for BE and other GERD complications, which could confound the analysis. Cohort 2 (GERD only), which included patients of the same age group diagnosed with GERD but without any evidence of current or past *H. pylori* infection (a negative laboratory result (e.g., urea breath test, stool antigen, or serum antibody)). The same exclusion criteria were applied.

The index event was defined as the first occurrence of GERD (with or without *H. pylori*), and patients were followed starting one day after the index date. The follow-up period was open-ended, limited only by the availability of clinical records within the 20-year lookback window.

Study analysis

To control for confounding, 1:1 propensity score matching (PSM) was performed using nearest-neighbor matching without replacement. Matching variables included age at index, sex, race, obesity, and personal history of nicotine dependence. After matching, each cohort contained 179,757 patients with balanced baseline characteristics (standardized differences < 0.001 for all variables).

The primary outcome was the incidence of BE (ICD-10: K22.7) [10]. Outcome measures included absolute risk, risk difference, risk ratio (RR), and odds ratio (OR) with 95% confidence intervals (CIs). Kaplan-Meier survival analysis was used to estimate cumulative incidence over time, and the log-rank test was used to compare survival distributions between cohorts. Hazard ratios (HR) were calculated using Cox proportional hazards models. Additionally, the number of BE diagnosis instances per patient was assessed based on patient visits. Only patients with at least one outcome event were included in this analysis. Descriptive statistics, including mean, standard deviation, and median, were calculated, and comparisons between cohorts were made using independent t-tests.

All statistical analyses were conducted using the TriNetX analytics platform [9]. Since the data were de-identified and fully compliant with the Health Insurance Portability and Accountability Act (HIPAA), the study was exempt from institutional review board (IRB) approval.

## Results

A total of 359,514 patients met the inclusion criteria and were included in the final analysis, with 179,757 patients in each cohort following 1:1 propensity score matching. Cohort 1 consisted of patients with GERD and documented *H. pylori* infection, while Cohort 2 included patients with GERD but no history of *H. pylori*.

Prior to matching, significant differences were observed between the two cohorts across several demographic and clinical variables, including age, sex, race, obesity, and history of nicotine dependence. However, after matching, all baseline characteristics were well balanced, with standardized mean differences of less than 0.001 for all covariates. The mean age in both cohorts was 32.7 years, and approximately 65% of patients in each group were female. Racial distribution was comparable across both groups, with the majority of patients identifying as White (52.7%), followed by Black or African American (17.6%), Asian (6.5%), and other races.

**Figure 1:**
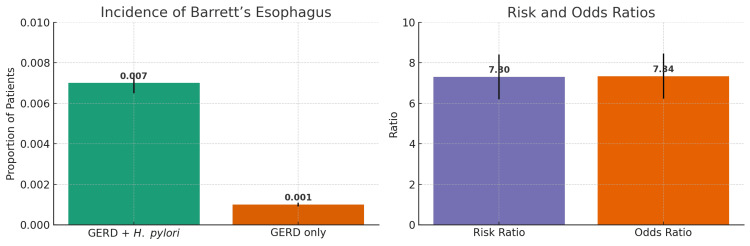
Incidence, risk ratio, and odds ratio of Barrett’s esophagus in GERD patients with and without H. pylori infection Left: Bar chart with error bars displaying the incidence of Barrett’s esophagus in the two cohorts (chi-square test, p-value < 0.0001); Right: Risk ratio and odds ratio comparing the GERD + *H. pylori* group to the GERD-only group, with approximate confidence intervals (chi-square test, p-value < 0.0001). GERD: gastroesophageal reflux disease; *H. pylori*: *Helicobacter pylori*

The incidence of BE was significantly higher among GERD patients with *H. pylori* infection. Specifically, 1,183 patients (0.66%) in the GERD + *H. pylori* cohort developed BE compared to 162 patients (0.09%) in the GERD-only cohort. The absolute risk of developing BE was 0.007 (95% CI: 0.0065-0.0074) in Cohort 1 and 0.001 (95% CI: 0.0009-0.0011) in Cohort 2 (Figure 1). The calculated risk difference was 0.006 (95% CI: 0.005-0.006), which was statistically significant with a p-value of less than 0.0001. The RR was 7.30 (95% CI: 6.20-8.60), and the OR was 7.34 (95% CI: 6.23-8.66), indicating that patients with GERD and *H. pylori* infection were over seven times more likely to be diagnosed with BE than those without *H. pylori* (Figure 1).

**Figure 2:**
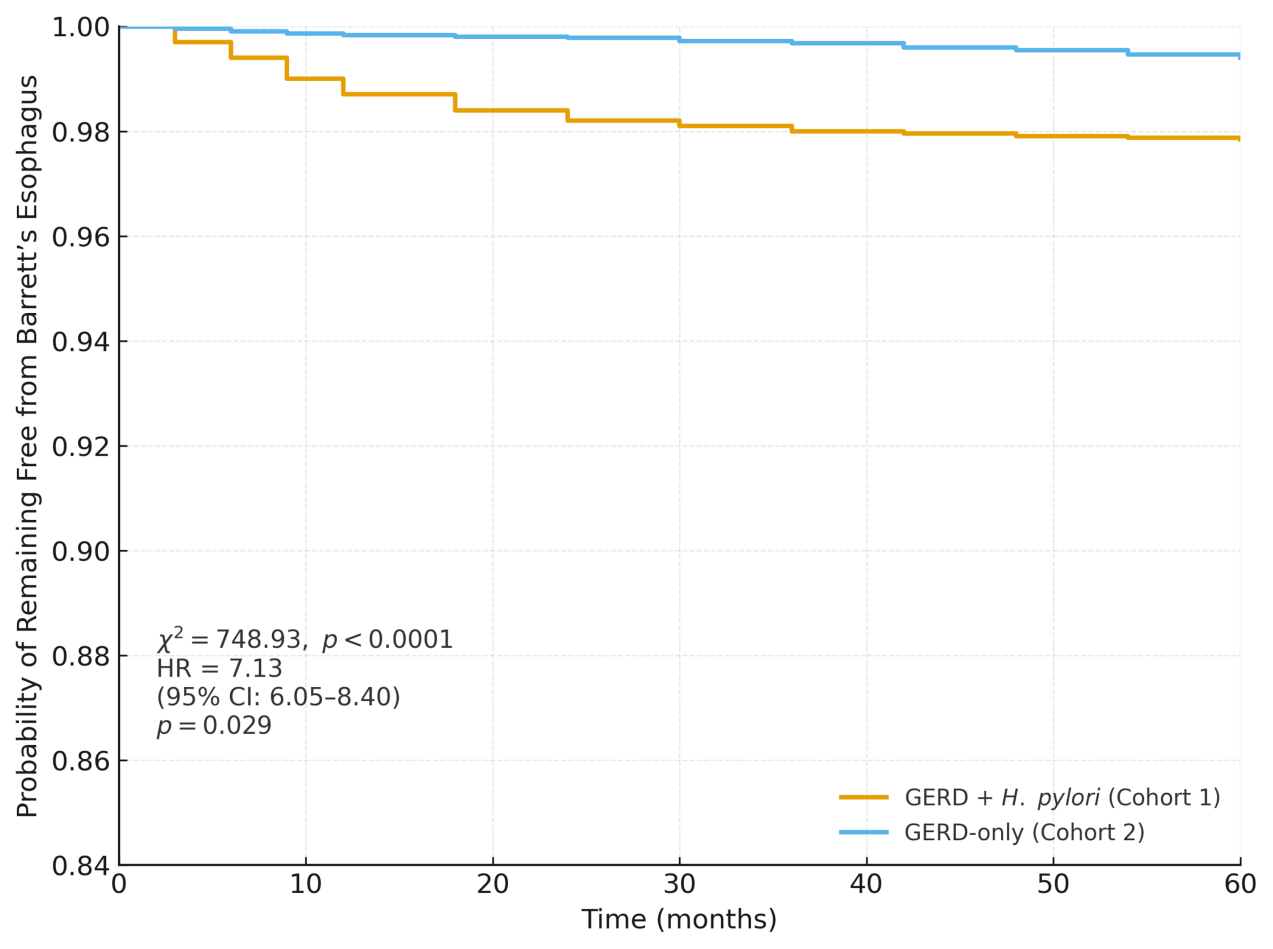
Kaplan-Meier survival curve comparing risk of Barrett’s esophagus Kaplan–Meier curve showing reduced survival in GERD + *H. pylori *patients, with a hazard ratio of 7.13 for developing Barrett’s esophagus. Log-rank test demonstrated statistical significance (p < 0.05). GERD: gastroesophageal reflux disease; *H. pylori*: *Helicobacter pylori*

Survival analysis using Kaplan-Meier curves further supported this association. The probability of remaining free from BE over the study period was significantly lower in the GERD + *H. pylori* cohort compared to the GERD-only cohort. At the end of the follow-up period, 97.84% of patients in Cohort 1 and 99.40% in Cohort 2 remained free of BE, indicating that Cohort 1 had a slightly higher risk of developing the condition. The log-rank test showed a highly significant difference in the risk of developing BE between the groups (chi-square (χ²) = 748.93, p < 0.0001), indicating that the timing of disease onset differed greatly. Specifically, patients in the GERD + *H. pylori* group had a much higher risk, with an HR of 7.13 (95% CI: 6.05-8.40, p = 0.029), meaning they were over seven times more likely to develop BE compared to the reference group (Figure 2).

Additionally, the analysis of the number of diagnosis instances revealed a higher recurrence or follow-up rate of BE among GERD patients with *H. pylori*. Patients in Cohort 1 had a mean of 3.15 ± 4.65 diagnosis instances, while those in Cohort 2 had a mean of 1.67 ± 1.67. The median number of instances was two in Cohort 1 and 1 in Cohort 2. A t-test comparing the two groups yielded a statistically significant result (t = 4.035, df = 1343, p < 0.0001), further suggesting more frequent documentation or recurrence of BE in the *H. pylori*-positive group.

## Discussion

Our study found a significantly higher incidence of BE among GERD patients with *H. pylori* infection compared to those without. Patients in the GERD + *H. pylori *cohort had an absolute risk of 0.007 versus 0.001 in the GERD-only cohort, with an RR of 7.30 and an OR of 7.34, indicating over a sevenfold increased likelihood of developing BE. Kaplan-Meier survival analysis reinforced this association, showing a lower probability of remaining free from BE in the *H. pylori*-positive group (97.84% vs. 99.40%), with a significant HR of 7.13 (95% CI: 6.05-8.40). Furthermore, the *H. pylori* group exhibited a higher frequency of diagnosis instances, suggesting more frequent recurrence or follow-up documentation. These findings highlight a strong temporal and statistical association between *H. pylori* infection and increased risk of BE in GERD patients. Importantly, although the RRs were high, the absolute risk difference between the two groups was modest, reflecting the influence of the large sample size on statistical significance.

The observed association between *H. pylori* infection and an increased risk of BE in GERD patients contrasts with earlier studies suggesting a protective role of *H. pylori*, particularly cagA-positive strains, against esophageal metaplasia [7,8]. While some research has proposed that *H. pylori*-induced hypochlorhydria might mitigate GERD severity and subsequent BE development, our findings align with emerging evidence indicating a more complex, context-dependent relationship. For instance, a meta-analysis by Fischbach et al. (2012) found no significant protective effect of *H. pylori* against BE, and certain strains may instead promote inflammatory cascades that exacerbate mucosal damage [11]. Similarly, a cohort study by Sonnenberg et al. (2010) reported that *H. pylori* infection was associated with a higher prevalence of GERD complications, including BE, particularly in younger populations [12]. Our results, derived from a large, matched cohort, reinforce the possibility that *H. pylori* may act as a cofactor in esophageal injury, particularly in the setting of preexisting GERD. Mechanistically, *H. pylori* infection can contribute to chronic inflammation and disrupt the gastric microbiome, potentially leading to increased acid-mediated mucosal injury. This may exacerbate the reflux-induced damage in GERD patients, promoting the development of BE through increased epithelial turnover, oxidative stress, and local cytokine-mediated injury.

Other studies have also explored the relationship between *H. pylori *and esophageal conditions with mixed outcomes. A population-based study by El-Serag et al. suggested an inverse association between *H. pylori* and BE, particularly among patients with gastric atrophy, implying a protective role via reduced acid output [13]. In contrast, a study by Thrift et al. found no statistically significant association between *H. pylori *infection and risk of BE after adjusting for confounders such as age, BMI, and smoking history [14]. Additionally, a systematic review by Rokkas et al. concluded that while some subtypes of *H. pylori* may confer protection against GERD and its complications, the overall evidence remains inconsistent due to geographic, demographic, and methodological variations [15]. These divergent findings underscore the complexity of the interaction between *H. pylori*, gastric physiology, and esophageal disease, reinforcing the need for large-scale, well-matched studies like ours to better delineate causality and clinical significance.

The clinical implications of our findings are significant, as they challenge the traditional view of *H. pylori*, a solely protective entity in esophageal pathology. Given the sevenfold increased risk of BE in *H. pylori*-positive GERD patients, targeted surveillance strategies may be warranted for this subgroup, especially younger adults who might otherwise be considered lower risk. Prior guidelines have not explicitly addressed *H. pylori* screening in GERD patients for BE risk stratification, but our data suggest that such an approach could improve early detection [2,16]. Additionally, the higher frequency of BE diagnosis instances in the *H. pylori *cohort raises questions about whether infection contributes to more aggressive disease progression or simply greater clinical scrutiny. A study by Rubenstein et al. (2014) noted that *H. pylori*-associated gastritis might alter reflux composition, potentially accelerating metaplastic changes [17]. Further mechanistic studies are needed to clarify whether *H. pylori* eradication modifies BE risk, but our findings underscore the importance of recognizing *H. pylori* as a potential risk modifier in GERD management.

This study leveraged a large, multicenter, real-world dataset from the TriNetX network [9], enhancing generalizability and statistical power. Propensity score matching minimized confounding by balancing key covariates across cohorts, and the use of multiple outcome measures, including absolute risk, risk ratios, and Kaplan-Meier survival curves, strengthened the robustness of the findings. However, limitations include the retrospective design and reliance on administrative ICD-10 coding [10], which may introduce misclassification bias. Additionally, data on endoscopic findings, treatment compliance, and strain-specific information for *H. pylori *were unavailable, potentially affecting causal interpretation. Furthermore, we acknowledge the possibility of residual confounding from unmeasured variables such as medication use (including proton pump inhibitors and antibiotics), GERD severity or duration, and smoking intensity, which may have influenced the observed associations. As this is a retrospective observational study, causality cannot be established, and the findings should be interpreted as associations rather than causal relationships.

## Conclusions

In this large retrospective cohort study, we found a significant association between *H. pylori* infection and increased risk of BE among patients with GERD. Patients with concurrent GERD and *H. pylori *infection were over seven times more likely to develop BE compared to GERD patients without the infection. These findings challenge previous assumptions of a protective role of *H. pylori *and suggest that, in certain contexts, the bacterium may act as a cofactor in esophageal metaplasia. The increased number of BE diagnosis instances in the *H. pylori*-positive group may indicate more severe or recurrent disease, although further studies are needed to explore causality and biological mechanisms. Clinically, our results support the potential benefit of considering *H. pylori* status in risk stratification and surveillance planning for GERD patients. Future prospective research should examine whether eradication of *H. pylori* alters the natural history of BE development.
